# Supplementary material for: Drug Utilization and Measurement of Medication Adherence: A Real World Study of Psoriasis in Italy
Source: Pharmaceutics. 2023 Nov 21;15(12):2647. doi: 10.3390/pharmaceutics15122647 (PMC10747905; doi:10.3390/pharmaceutics15122647)

Supplementary Material

Figure S1. Schematic presentation of PSO patients' selection

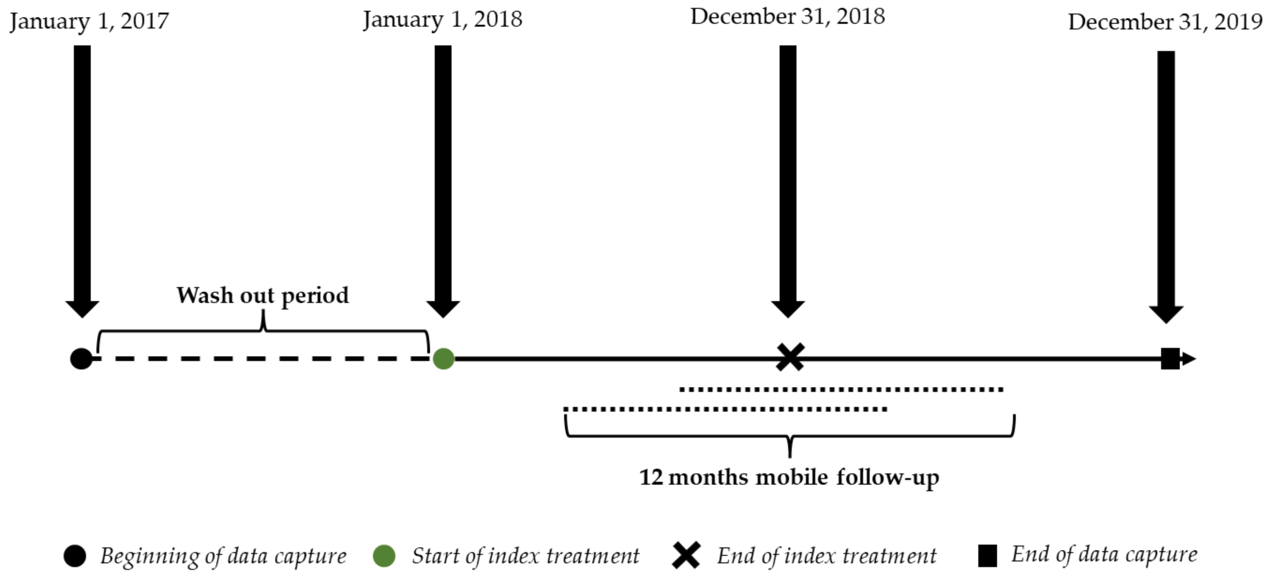

Figure S2. Study flowchart

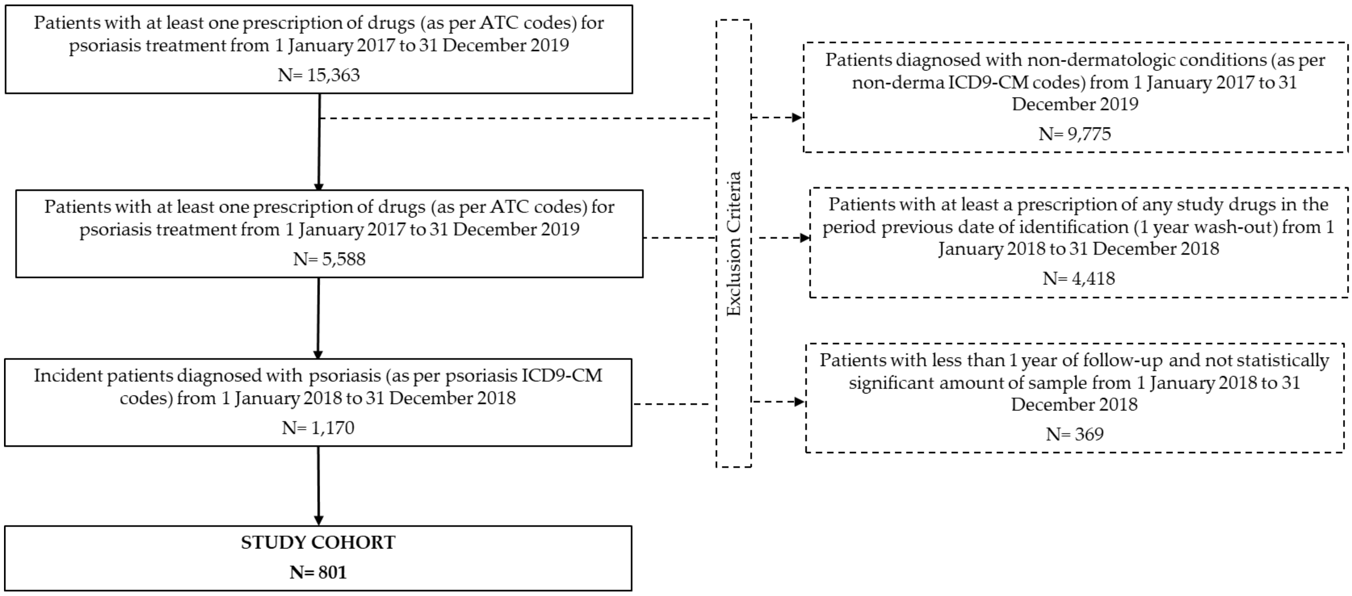

Table S1. Overview of study drugs charateristics

ATC	Active Substance	Trade Name	Posology	Drug speciality code	Year of Marketing Authorisation	Therapeutic Indications	Reimbursability	Ex Factory Price	Additional Monitoring
**D05 Antipsoriatics**									
**D05BB02**	**Acitretin**	**Neotigason**	25 mg/20 tablets	027480021	26/04/1997	Severe forms of psoriasis, pustular psoriasis,Severe and refractory forms of dermatitis, Darier's disease lichen planus	A	19,83 €	Additional Monitoring
			10 mg/30 tablets	027480019			A	14,11 €	Additional Monitoring
		**Zorias**	25 mg/20 tablets	041619065	feb-13		A	12,73 €	Additional Monitoring
			10 mg/30 tablets s	041619014			A	9,06 €	Additional Monitoring
**L04 Immunosuppressants**									
**L04AD01**	**Cyclosporine**	**Ciqorin**	100 mg/ 30 tablets	042787299	-	Organ transplantation, Bone marrow transplantation, Endogenous uveitis, Nephrotic syndrome, Rheumatoid arthritis, Psoriasis, Atopic dermatitis	A	€ 29,52	-
			10mg/ 50 tablets	042787251			A	€ 5,42	-
			25mg/ 50 tablets	042787263			A	€ 13,55	-
			50mg/ 50 tablets	042787275			A	€ 26,27	-
**L04AX03**	**Methotrexato**	**Reumaflex**	10mg/ 0,2 ml	039153123	29/12/2009	Rheumatoid arthritis, Juvenile idiopathic arthritis, Severe psoriasis, Crohn's disease	A	€ 17,85	-
			12,5mg/0,25ml	039153628	29/12/2009		A	€ 18,20	-
			15mg/0,30ml	039153224			A	€ 26,07	-
			17,5mg/0,35ml	039153679			A	€ 25,09	-
			20mg/0,40ml	039153325			A	€ 34,48	-
			25mg/0,50ml	039153426			A	€ 42,34	-

**L04AX07**	**Dimethylfumarate**	**Skilarence**	30 mg/42 tablets	045493018	23/06/2017	Psoriasis plaques	A	94,16 €	-
			120 mg/ 90 tablets	045493044			A	201,78 €	-
**L04AA29**	**Tofacitinib**	**Xeljanz**	50mg/56 tables	045320037	22/03/2017	Rheumatoid arthritis, Psoriatic arthritis, Ankylosing spondylitis, Ulcerative colitis, Juvenile idiopathic arthritis	H	720,92 €	AIFA Note 06/07/2021
			50mg/182 tablets	045320049			H	2.342,83 €	
			50mg/112 tablets	045320102			H	1.441,83 €	
			10mg/112 tablets	045320088			H	2.883,67 €	
**L04AA32**	**Apremilast**	**Otezla**	30mg/56 tablets	043867023	15/01/2015	Psoriatic arthritis, Psoriasis, Behçet's disease	A	758,10 €	Additional Monitoring
			4 x 10 mg + 4 x 20 mg + 19 x 30 mg tablets	043867011			A	365,51 €	
**L04AB01**	**Etanercept**	**Benepali**	25mg/0,51ml 4syringes	044691057	14/01/2016	Rheumatoid arthritis, Juvenile idiopathic arthritis, Psoriatic arthritis, Axial spondyloarthritis, Plaque psoriasis, Paediatric plaque psoriasis	H	314,07 €	Additional Monitoring – AIFA paper form
			50mg/1ml 4 syringes	044691018			H	629,00 €	
			50mg/4 syringes	044691020			H	629,00 €	
		**Erelzi**	25mg/0,5ml 4 syringes	045451034	23/06/2017		H	299,34 €	Additional Monitoring – AIFA paper form
			50mg/1ml 4 syringes	045451073			H	598,66 €	
			50mg/1ml 4 pens	045451111			H	598,66 €	
		**Enbrel**	10 mg 1 vial	034675215	03/02/2000		H	184,21 €	AIFA paper form
			25 mg 4 syringes	034675102			H	460,51 €	

			50 mg 1 ml 4 pens	034675191			H	921,08 €	
			25 mg 4 syringes	034675037			H	460,51 €	
			50 mg 4 syringes	034675140			H	921,02 €	
L04AB02	Infliximab	**Remicade**	100mg 1 vial	034528012	13/08/1999	Rheumatoid arthritis, Crohn's disease, Ulcerative colitis, Ankylosing spondylitis, Psoriatic arthritis, Psoriasis	H	463,53 €	AIFA paper form - AIFA note 07/03/2022
		**Zessly**	100mg 1 vial	046635013	18/05/2018		H	386,28 €	Additional Monitoring – AIFA paper form - AIFA note 07/03/2022
		**Flixabi**	100mg 1 vial	044892014	26/05/2016		H	386,28 €	
L04AB02	Infliximab	**Inflectra**	100mg 1 vial	043010014	10/09/2013	Rheumatoid arthritis, Crohn's disease, Ulcerative colitis, Ankylosing spondylitis, Psoriatic arthritis, Psoriasis	H	386,28 €	AIFA Note 07/03/2022
		**Remsima**	100mg 1 vial	042942019	22/11/2019		H	386,28 €	AIFA paper form - AIFA note 07/03/2022
			120mg/ml 1 pen	042942122			H	288,98 €	
			120mg/ml 4 pens	042942146			H	1.155,94 €	
L04AB04	Adalimumab	**Amgevita**	20mg/0,4ml 1 syringe	045317017	22/03/2017	Rheumatoid arthritis, Juvenile idiopathic arthritis, Axial spondyloarthritis, Psoriatic arthritis, Paediatric plaque psoriasis, Crohn's disease, Ulcerative colitis, Uveitis	H	192,87 €	AIFA paper form
			40mg/0,8ml 2 pens	045317070			H	771,50 €	
			40mg/0,8ml 2 syringes	045317031			H	771,50 €	
		**Humira**	80mg/0,8ml 1 pen	035946209	08/09/2003		H	964,37 €	AIFA paper form
			80mg/0,8ml 1 syringe	035946197			H	964,37 €	
			40mg/0,4ml 2 pens	035946161			H	964,37 €	
		**Idacio**	40mg/0,8ml 1vial	047805015	02/04/2019		H	342,45 €	Additional Monitoring – AIFA paper form

			40mg/0,8ml 2pens	047805039			H	684,71 €	
			40mg/0,8ml 2 syringes	047805027			H	684,71 €	
		Yuflyma	40mg/0,4ml 1 pen	049376092	11/02/2021		H	342,35 €	Additional Monitoring – AIFA paper form
			40mg/ 0,4ml 4 pens	049376116			H	1.369,42 €	
		Hyrimoz	40mg/0,8ml 2 pens	046889059	26/07/2018		H	684,71 €	Additional Monitoring – AIFA paper form
			20mg/04ml 2 syringes	046889073			H	342,35 €	
			40mg/0,8ml 2 pens	046889022			H	684,71 €	
		Imraldi	40mg/0,8ml 2 pens	045616063	24/08/2017		H	684,71 €	Additional Monitoring – AIFA paper form
			40mg/0,8ml 2 syringes	045616024			H	684,71 €	
**L04AB05**	**Certolizumab**	**Cimzia**	200mg/ml 1 syringe	039539085	01/10/2009	Rheumatoid arthritis, Axial spondyloarthritis, Ankylosing spondylitis, Psoriasis plaques, Psoriatic arthritis	H	920,55 €	AIFA paper form
			200mg/ml 2 pens	039539059			H	920,55 €	
			200mg/ml 2 siringhe	039539010			H	920,55 €	
**L04AC05**	**Ustekinumab**	**Stelara**	130 mg 26 ml 1 vial	038936050	16/01/2009	Psoriasis plaques, Psoriatic arthritis, Crohn's disease, Ulcerative colitis	H	2.842,88 €	AIFA paper form
			45 mg/0,5 ml 1 syringe	038936035			H	2.842,88 €	
			90 mg/ml 1 syringe	038936047			H	2.842,88 €	

			45 mg/0,5 ml 1 vial	038936011			H	2.842,88 €	
**L04AC10**	**Secukinumab**	**Cosentyx**	150 mg/ml 1 pen	043873049	15/01/2015	Plaque psoriasis in paediatric patients juvenile idiopathic arthritis, Arthritis related to enthesitis, Juvenile psoriatic arthritis	H	473,81 €	AIFA paper form
			300 mg/2 ml pens	043873102			H	947,63 €	
			150 mg/ ml 1 syringe	043873025			H	473,81 €	
			300 mg/2 ml 150 mg/ml 1 syringe	043873088			H	947,63 €	
			150 mg/ ml 2 pens	043873052			H	947,63 €	
			150 mg/ ml 2 syringes	043873037			H	947,63 €	
**L04AC12**	**Brodalumab**	**Kyntheum**	1,5 ml 140 mg/ml 2 syringes	045484019	17/07/2017	Psoriasis plaques	H	1.105,36 €	Additional Monitoring – AIFA paper form
**L04AC13**	**Ixekizumab**	**Taltz**	80/ ml mg 2 pens	044863025	25/04/2016	Psoriasis plaques, Paediatric plaque psoriasis, Psoriatic arthritis, Axial spondylarthritis	H	2.025,40 €	-
			80/ ml mg 2 syringes	044863052			H	2.025,40 €	
**L04AC16**	**Guselkumab**	**Tremfya**	100 mg/ml 1 ml 1 pen	045772023	10/11/2017	Psoriatic arthritis, Psoriasis plaques	H	2.062,98 €	Additional Monitoring – AIFA paper form
			100 mg/ml 1 ml 1 syringe	045772011			H	2.062,98 €	
**L04AC17**	**Tildrakizumab**	**Ilumetri**	100mg/ml	047196011	17/09/2018	Psoriasis plaques	H	3.118,45 €	Additional Monitoring – AIFA paper form
**L04AC18**	**Risankizumab**	**Skyrizi**	75 mg/0,83 ml	047821018	26/04/2019	Psoriatic arthritis, Psoriasis plaques	H	3.230,00 €	Additional Monitoring – AIFA paper form

**L04AA56**	**Deucravacitinib**	-	-	-	-	Psoriatic arthritis, Psoriasis plaques	Not commercially available	-	-
**L04AC21**	**Bimekizumab**	**Bimzelx**	160mg/ml	-	-	Psoriasis plaques	CNN	-	Additional Monitoring – AIFA paper form
**L04AC21**	**Spesolimab**	**Spevigo**	450 mg	050464015	-	Psoriasis pustularis	CNN	-	-

Abbreviations: A: essential drugs and those for chronic diseases, fully reimbursed by the National Health Service (NHS); H: drugs for hospital use only that can be used only in hospitals and/or provided in the context of hospitals and assimilated facilities; CNN: band c, non-negotiated; Aifa notes: regulatory instrument that defines the therapeutic indications for which a particular drug is reimbursable under the NHS; Additional Monitoring: Medicines subject to close and specific monitoring by regulatory agencies.

Table S2. Description of induction and maintenance phases of PSO pharmacology therapy

ATC V	Active Substance	Drug speciality	Drug speciality Description	Package Days	Induction Posology Description	Maintenance Posology Description
L04AA32	Apremilast	043867011	4cpr x 10mg<br>4cpr x 20mg<br>19cpr x 30mg tablets	**14**	1 tablet 10mg daily 1, 2 tablets 10mg daily 2, 1 tablet 10mg and 1 tablet 20mg daily 3, 2 tablets 20mg daily 4, 1 tablet 20mg and 1 tablet 30mg daily 5, 2 tablets 30mg daily for 9 days	-
		043867023	30 mg 56 tablets	**-**	-	2 tablets every day for as long as the Therapeutic Plan is valid
L04AB01	Etanercept	04675052	50mg/1ml<br>4 syringes	**14**	2 x 50mg syringes every 7 days for 84 days	1 x 50 mg syringe every 7 days for as long as the Therapeutic Plan lasts
		034675013	50mg/1ml<br>4 syringes	**14**	2 x 50mg syringes every 7 days for 84 days	1 x 50 mg syringe every 7 days for as long as the Therapeutic Plan lasts
		034675037	25mg/1ml<br>4 syringes	**7**	2 x 50mg syringes every 7 days for 84 days	1 x 50 mg syringe every 7 days for as long as the Therapeutic Plan lasts
		034675102	25mg/0,5ml<br>4 syringes	**7**	2 x 50mg syringes every 7 days for 84 days	1 x 50 mg syringe every 7 days for as long as the Therapeutic Plan lasts
		034675140	50mg/1ml<br>4 syringes	**14**	2 x 50mg syringes every 7 days for 84 days	1 x 50 mg syringe every 7 days for as long as the Therapeutic Plan lasts
		034675191	50mg/1ml<br>4 pens	**14**	2 pens of 50mg every 7 days for 84 days	1 pen of 50 mg every 7 days for as long as the Therapeutic Plan lasts
		044691020	50mg/1ml<br>4 pens	**14**	2 pens of 50mg every 7 days for 84 days	1 pen of 50 mg every 7 days for as long as the Therapeutic Plan lasts
	Etanercept Biosimilare	044691018	50mg/1ml<br>4 syringes	**14**	2 x 50mg syringes every 7 days for 84 days	1 x 50 mg syringe every 7 days for as long as the Therapeutic Plan lasts
		044691057	50mg/1ml<br>4 syringes	**14**	2 x 50mg syringes every 7 days for 84 days	1 x 50 mg syringe every 7 days for as long as the Therapeutic Plan lasts

L04AB04	Adalimumab	035946019	40mg/0,8ml 2 syringes	**11**	2 syringes of 40mg on the first day and 1 syringe after 7 day	1 syringe every 14 days for as long as the Therapeutic Plan is valid
		035946209	80mg/0,8ml 1 pen	**7**	2 pens of 40mg on the first day and 1 pen after 7 day	1 pen every 14 days for as long as the Therapeutic Plan is valid
		035946033	40mg/0,8ml 2 syringes	**11**	2 syringes of 40mg on the first day and 1 syringe after 7 day	1 syringe every 14 days for as long as the Therapeutic Plan is valid
		035946084	40mg/0,8ml 2 pens	**11**	2 pens of 40mg on the first day and 1 pen after 7 days	1 pen every 14 days for as long as the Therapeutic Plan is valid
		035946122	40mg/0,4ml 2 syringes	**11**	2 syringes of 40mg on the first day and 1 syringe after 7 day	1 syringe every 14 days for as long as the Therapeutic Plan is valid
		035946161	40mg/0,4ml 2 pens	**11**	2 pens of 40mg on the first day and 1 pen after 7 days	1 pen every 14 days for as long as the Therapeutic Plan is valid
	Adalimumab Biosimilare	045317031	40mg/0,8ml 2 syringes	**11**	2 syringes of 40mg on the first day and 1 syringe after 7 day	1 syringe every 14 days for as long as the Therapeutic Plan is valid
		046889022	40mg/0,8ml 2 pens	**11**	2 pens of 40mg on the first day and 1 pen after 7 days	1 pen every 14 days for as long as the Therapeutic Plan is valid
		047805027	40mg/0,8ml 2 syringes	**11**	2 syringes of 40mg on the first day and 1 syringe after 7 day	1 syringe every 14 days for as long as the Therapeutic Plan is valid
		045616024	40mg/0,8ml 2 syringes	**11**	2 syringes of 40mg on the first day and 1 syringe after 7 day	1 syringe every 14 days for as long as the Therapeutic Plan is valid
L04AB05	Certolizumab	039539010	200mg/ml 2 syringes	**14**	2 syringes every 14 days for 42 days	1 syringe every 14 days for as long as the Therapeutic Plan lasts
		039539059	200mg/ml 2 pens	**14**	2 pens every 14 days for 42 days	1 syringe every 14 days for as long as the Therapeutic Plan lasts
L04AC05	Ustekinumab*	038936023	90mg/ml 1 vial	**28**	1 injection every 28 days for 56 days	1 injection every 84 days for as long as the Therapeutic Plan lasts
		038936011	45mg/0,5ml 1 vial	**28**	1 injection every 28 days for 56 days	1 injection every 84 days for as long as the Therapeutic Plan lasts
		038936035	45mg/0,5ml 1 syringe	**28**	1 syringe every 28 days for 56 days	1 syringe every 84 days for as long as the Therapeutic Plan lasts
		038936047	90mg/ml 1 syringe	**28**	1 syringe every 28 days for 56 days	1 syringe every 84 days for as long as the Therapeutic Plan lasts

L04AC10	Secukinumab	043873025	150mg/ml 1 syringe	**4**	2 syringes every 7 days for 35 days	2 syringes every 28 days for as long as the Therapeutic Plan lasts
		043873102	300mg/2ml 1 pen	**7**	1 pens every 7 days for 35 days	1 pen every 28 days for as long as the Therapeutic Plan lasts
		043873037	150mg/ml 2 syringes	**7**	2 syringes every 7 days for 35 days	2 syringes every 28 days for as long as the Therapeutic Plan lasts
		043873049	150mg/ml 1 pen	**4**	2 pens every 7 days for 35 days	2 pens every 28 days for as long as the Therapeutic Plan lasts
		043873052	150mg/ml 2 pens	**7**	2 pens every 7 days for 35 days	2 pens every 28 days for as long as the Therapeutic Plan lasts
**L04AC12**	**Brodalumab**	045484019	210mg 2 syringes	**18**	1 syringe every 7 days for 21 days	1 syringe every 14 days for as long as the Therapeutic Plan lasts
**L04AC13**	**Ixekizumab**	044863025	80mg/ml 2 syringes	**25**	2 syringes every 14 days and 1 syringe every 14 days for 84 days	1 syringe every 28 days for as long as the Therapeutic Plan lasts
		044863052	80mg/ml 2 syringes	**25**	2 syringes every 14 days and 1 syringe every 14 days for 84 days	1 syringe every 28 days for as long as the Therapeutic Plan lasts
		044863013	80mg/ml 1 pen	**13**	2 pens every 14 days and 1 syringe every 14 days for 84 days	1 pen every 28 days for as long as the Therapeutic Plan lasts
**L04AC16**	**Guselkumab**	045772011	100mg/l 1 syringe	**28**	1 syringe every 28 days for 56 days	1 syringe every 56 days for as long as the Therapeutic Plan lasts
**L04AC17**	**Tildrakizumab**	047196011	100mg/ml 1 syringe	**28**	1 syringe every 28 days for 56 days	1 syringe every 84 days for as long as the Therapeutic Plan lasts
**L04AC18**	**Risankizumab**	047821018	75mg/0,83ml 2 syringes	**28**	2 syringes every 28 days for 56 days	2 syringes every 84 days for as long as the Therapeutic Plan lasts

** ustekinumab 45 mg if body weight is between 60 kg and 100 kg, ustekinumab 90 mg if weight > 100*

Table S3. Switch and Swap therapy among drug classes for PSO treatment

		Anti- PDE4	Anti TNF-α				Anti IL-12/23	Anti IL-17			Anti IL-23		
		Apremilast	Etanercept	Infliximab	Adalimumab	Certolizumab	Ustekinumab	Secukinumab	Brodalumab	Ixekizumab	Guselkumab	Tildrakizumab	Risankizumab
Anti- PDE4	Apremilast		Swap	Swap	Swap	Swap	Swap	Swap	Swap	Swap	Swap	Swap	Swap
Anti TNF-α	Etanercept	Swap		Switch	Switch	Switch	Swap	Swap	Swap	Swap	Swap	Swap	Swap
	Infliximab	Swap	Switch		Switch	Switch	Swap	Swap	Swap	Swap	Swap	Swap	Swap
	Adalimumab	Swap	Switch	Switch		Switch	Swap	Swap	Swap	Swap	Swap	Swap	Swap
	Certolizumab	Swap	Switch	Switch	Switch		Swap	Swap	Swap	Swap	Swap	Swap	Swap
Anti IL-12/23	Ustekinumab	Swap	Swap	Swap	Swap	Swap		Swap	Swap	Swap	Swap	Swap	Swap
Anti IL-17	Secukinumab	Swap	Swap	Swap	Swap	Swap	Swap		Switch	Switch	Swap	Swap	Swap
	Brodalumab	Swap	Swap	Swap	Swap	Swap	Swap	Switch		Switch	Swap	Swap	Swap
	Ixekizumab	Swap	Swap	Swap	Swap	Swap	Swap	Switch	Switch		Swap	Swap	Swap
Anti IL-23	Guselkumab	Swap	Swap	Swap	Swap	Swap	Swap	Swap	Swap	Swap		Switch	Switch
	Tildrakizumab	Swap	Swap	Swap	Swap	Swap	Swap	Swap	Swap	Swap	Switch		Switch
	Risankizumab	Swap	Swap	Swap	Swap	Swap	Swap	Swap	Swap	Swap	Switch	Switch	

Legend			
Indicator	Drug class	Swap	Switch
▲	Anti- PDE4	▲	▲
▲	Anti TNF-α	▲	▲
▲	Anti IL-12/23	▲	▲
▲	Anti IL-17	▲	▲

Table S4. Pattern swaps and switches between drugs for the treatment of psoriasis

			Anti- PDE4	Anti TNF-α		Anti IL-12/23	Anti IL-17		
		Overall	Apremilast	Etanercept	Adalimumab	Ustekinumab	Secukinumab	Brodalumab	Ixekizumab
Anti- PDE4	Apremilast	165	141	4	1	4	3	1	10
Anti TNF-α	Etanercept	56	2	37	1	8	5		3
	Infliximab	1							
	Adalimumab	132	1	2	114	4	5		5
	Certolizumab	9	1				1		2
Anti IL-12/23	Ustekinumab	124	3	1	3	109			7
Anti IL-17	Secukinumab	183	4		3	4	161		5
	Brodalumab	0						0	
	Ixekizumab	141		1	2	4	2		128

Legend			
Indicator	Drug class	Swap	Switch
▲	Anti- PDE4	▲	▲
▲	Anti TNF-α	▲	▲
▲	Anti IL-12/23	▲	▲
▲	Anti IL-17	▲	▲

Figure S3. One year medication persistence rates to PSO treatments (30 days gap)

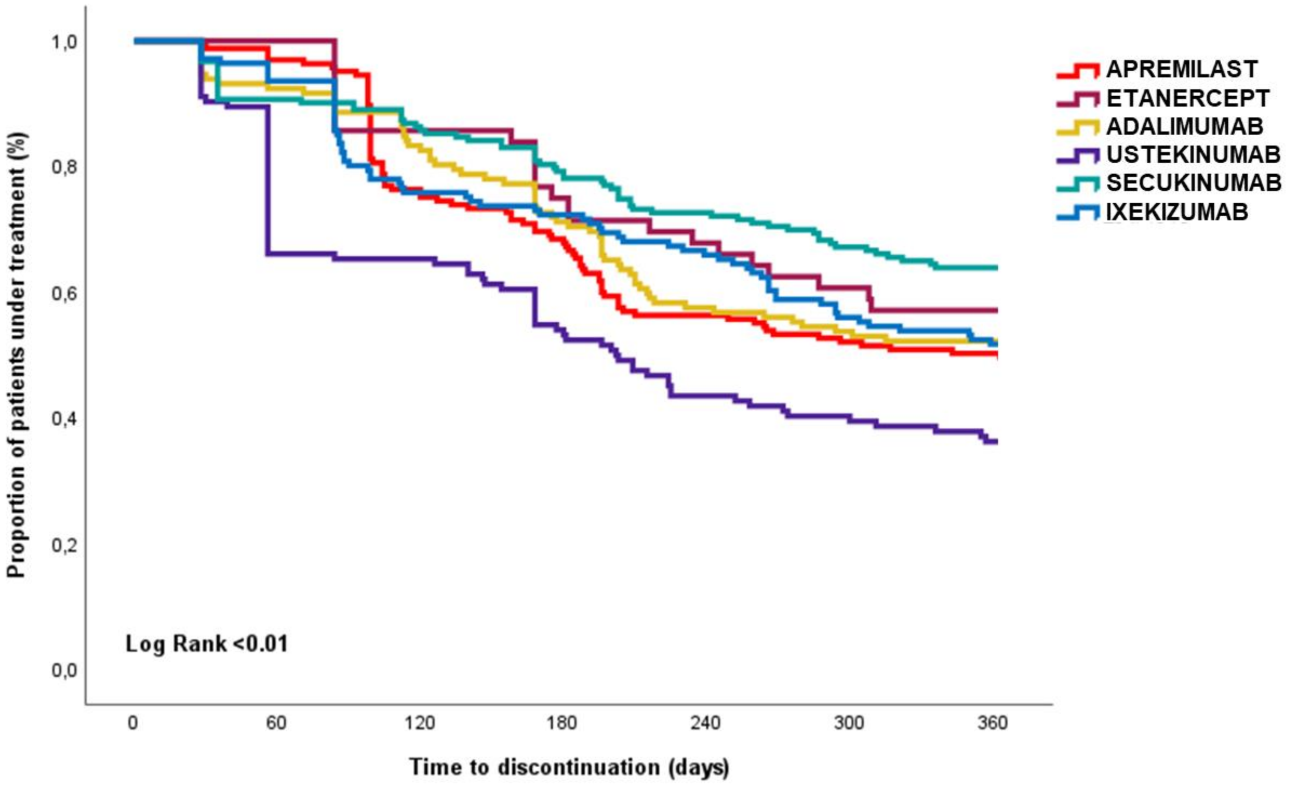

Figure S4. One year medication persistence rates to PSO treatments (90 days gap)

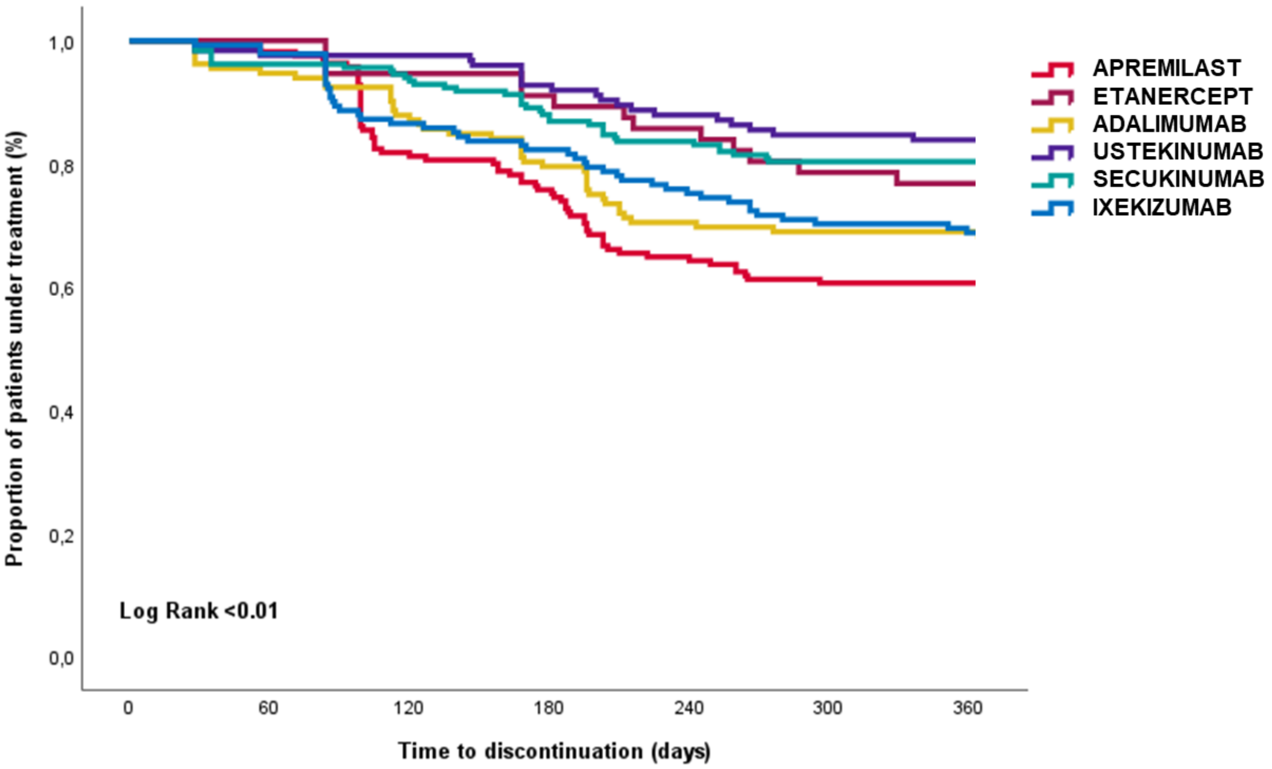

Supplement: Supplementary file 1 [file pharmaceutics-15-02647-s001.zip › pharmaceutics-2688676-SI.pdf]
